# Gender Differences in the Impact of Intergenerational Support on Depressive Symptoms among Older Adults in Korea

**DOI:** 10.3390/ijerph17124380

**Published:** 2020-06-18

**Authors:** Kyungwon Choi, Gyeong-Suk Jeon, Kwang-Sim Jang

**Affiliations:** 1 Department of Nursing, Korea National University of Transportation, Chungbuk 27909, Korea; kwchoi@ut.ac.kr; 2 Department of Nursing, Division of Natural Science, Mokpo National University, Muan-gun 58554, Korea; 3 Department of Nursing, Sehan University, Youngam 58447, Korea; ksjang@sehan.ac.kr

**Keywords:** depression, older adults, social support, intergenerational relations, gender

## Abstract

*Background*: This study examined the relationship between intergenerational support patterns and depressive symptoms among older men and women in Korea. *Methods*: A nationally representative survey of non-institutionalized, community-dwelling older adults in Korea was used. A total of 7531 older adults (3592 men and 3939 women) was included in the analysis. *Results*: We observed gender differences in the impact of financial support exchanges on depressive symptoms. A lack of mutual financial support significantly increased the risk of depressive symptoms by 3.83 times (95% CI 2.34–6.24) in men and 1.73 times (95% CI 1.06–2.83) in women. Men who received financial support were more likely to experience depressive symptoms (OR (Odds Ratio), 1.81, 95% CI 1.36–2.42), whereas women who provided financial support were more likely to experience depressive symptoms (OR 2.82, 95% CI 1.21–6.56). The lack of an exchange of emotional support was significantly associated with depressive symptoms in both men (OR 1.49, 95% CI 1.17–1.90) and women (OR 1.87, 95% CI 1.50–2.34). *Conclusions*: We discuss the evidence of gender differences in intergenerational support exchange patterns and their impact on depressive symptoms within the context of Korean cultures and suggest that future research should be conducted on gender differences in the impact of intergenerational support on mental health across diverse societies.

## 1. Introduction

The importance of intergenerational support for the psychological health of older adults is well-documented in the literature [1,2,3]. However, the impact of the exchange patterns of intergenerational support on psychological health in older adults is less clear. Some studies have indicated that older adults who receive more support show higher life satisfaction and psychological well-being [4], whereas others have reported that high levels of support from adult children is either harmful [5] or has a negligible effect on the well-being of older adults [6]. Recently, several studies have focused on the provider role of older parents, with some evidence suggesting that providing support to adult children improved the parents’ quality of life through maintaining social roles and increasing self-esteem [7,8,9]. From an equity theory perspective, several researchers have emphasized the importance of reciprocal support [10,11]. In these studies, older adults with balanced or reciprocal exchange patterns had better psychological outcomes compared to their counterparts with imbalanced patterns. These inconsistent findings indicate that further research is needed to determine the impact of intergenerational support patterns (providing, receiving or reciprocal) on psychological health.

In addition, there are considerable variations in the types of support and exchange patterns according to the cultural values and welfare regimes of the country [12,13]. For example, in Spain, being one of the most traditional family-centered societies, a high level of personal care was provided to older adults, while in Norway and Israel, with the development of public services, older adults relied less on their children for financial and caring support than those in Spain [12]. Thus, the support of adult children in these countries mainly takes the form of emotional and instrumental help [12,13]. Meanwhile, Eastern countries, such as Korea, China and Japan, have strong norms of family obligation and filial norms about caring for older parents. As a result, support for older persons involves the unidirectional dependency of older parents on adult children. Yoon [14] reported that 70.8% of Korean older parents received various kinds of support from their adult children. Moreover, in Korea, the public services available for older persons are still relatively underdeveloped. Neither public nor private pensions cover more than a third of those aged 65 and over. Korea’s basic pension scheme is not enough to guarantee economic security, and the Basic Livelihood Security Scheme has very low coverage [15]. Most life risks to people of older ages in Korea are still covered by family and kinship ties, especially through intergenerational relationships [16].

Researchers have suggested that older men and women differ in both quantitative and qualitative aspects of social support [5]. Gender differences in mutual intergenerational support have also been identified, with mothers being more likely to be involved in various types of intergenerational support exchange with their children compared to fathers [5,17]. Older women are more likely to depend on their children for financial support than older men [18], and they typically also receive and provide more emotional and instrumental support than older men [1,19]. Meanwhile, Silverstein and colleagues reported that instrumental support exchange mostly depends on the need and resources of older adults, regardless of gender [5]. Several recent studies have showed gender differences in the effects of intergenerational support exchange on psychological well-being [1,2,20]. Receiving emotional support was associated with improved mental health only in older women [2,20]; receiving instrumental and financial support was negatively associated with mental health in older men [1,2]. Providing instrumental support was positively associated with mental health in both older men and women [1,20], whereas providing financial support resulted in significantly improved psychological health in older men, but not in older women [1]. Li and colleagues found that mutual emotional support was more important to the subjective health of older women than unidirectional support [1]. Given these findings, the exchange patterns of intergenerational support and their impact on psychological health may differ by gender in older adults, since gender differences exist in various health outcomes, as well as in the degree of exposure to the social determinants of health [21], which are linked to gender-related social roles [22].

Differences in gender-related social roles and relations across countries may result in different gendered patterns of psychological health. In Korea, men and women often have clearly demarcated positions and roles within both their family and society under the influence of the Confucianism, the dominant ideology in the Chosŏn dynasty of Korea, dating back to the sixteenth century [23]. It has become a part of the foundation on which the moral values, way of life, gender roles and social relations between the old and young of contemporary Korean society are based [24]. While the modernization and westernization of Korean society have weakened these cultural norms, many older adults have maintained their traditional gendered social roles. Therefore, this study examines gender differences in intergenerational support patterns and their impact on depression among older adults in Korea, who have lived their entire lives within a gender-segregated society. We hypothesized that the exchange patterns of various types of intergenerational support, and their impact on the mental health of older adults in Korea, would differ by gender. The findings of this study may fill the knowledge gap regarding gender differences in intergenerational support exchange and their impact on depressive symptoms and could extend the understanding of cross-country variations.

## 2. Materials and Methods

### 2.1. Study Design and Population

Data from the Living Profiles of Older People Survey (LPOPS), conducted by the Korean Ministry of Health and Welfare in 2017, were analyzed in this study. The survey used a two-stage stratified cluster sampling method, with older residents being selected from households in 25 metropolitan and provincial (urban and rural) regions. Trained research staff visited participants at their places of residence and obtained informed consent. A total of 10,299 participants completed in-person interviews. Participants who had never married, and those without children, were excluded from the analysis. Adult children were sons or daughters of the participant who had attained the age of an adult (aged 18 and over). Since decreased mobility is closely related to the need for support [25], and may affect the types of intergenerational support provided and received, individuals with physical limitations were also excluded. Physical limitations were measured using the Korean version of the Instrumental Activities of Daily Living scale (K-IADL) [26]. The K-IADL includes 10 questions on the instrumental activities of daily living, such as personal grooming, short excursions, use of transportation, making and receiving phone calls, managing money, performing household chores, preparing meals, shopping, taking medication and doing laundry. Respondents who were dependent on others for one or more of the instrumental activities of daily living were considered to have a physical limitation. Ultimately, a weighted population of 7531 participants, aged 65 years and older, was included in the analysis.

### 2.2. Assessments and Measurements

Types and exchange patterns of intergenerational support. In older ages, most people face difficulty in terms of their finances and performing routine activities, and they can suffer from different kinds of disease and loneliness [12,27]. The primary support provider of these needs of older adults are family members such as adult children [28]. Therefore, this study employed four types of intergenerational support: whether the participants talked about their worries and troubles (emotional support); whether they received regular or irregular financial assistance (financial support); whether they had assistance with household tasks such as cleaning the house, washing clothes and preparing meals (instrumental support); and whether they received help with transportation to the hospital and were cared for when they were sick (caring support). The exchange patterns of support were derived from a set of items reflecting support exchange between adult children and older parents. To measure the amount of support provided by adult children, participants were asked about the frequency with which they received each kind of support: “How frequently do you receive support from your children?”. For each of these items, the response options were “not at all”, “rarely”, “often” and “usually”, which were categorized as “yes” (“often” and “usually”) or “no” (“not at all” and “rarely”) for analysis purposes. Then, with the same coding scheme, parallel questions were asked of the participants in order to ascertain whether the older parents provided each type of support to their adult children. Based on the questions about providing and receiving intergenerational support, the exchange patterns were categorized into four groups: no exchange, receiving support only, providing support only and mutual support. The first category of “no exchange” means there was no providing or receiving of each type of support between older parents and adult children. The second category of “receiving support only” indicated that older parents only received each type of support from their adult children, without providing it. The third category of “providing support only” indicated that older parents only provided each type of support to their adult children, without receiving it. The last category of “mutual support” indicated that older parents exchanged each type of support with their adult children.

Depressive symptoms. Depressive symptoms were evaluated using the Korean version of the Geriatric Depression Scale-Short Form (SGDS-K). The SGDS was originally developed by Yesavage and Sheik [29] and translated into Korean by Bae and Cho [30]. The SGDS-K is composed of 15 items taken from the 30-item GDS-K. The Korean versions of the GDS and SGDS are valid and widely used tools. The SGDS-K has shown satisfactory reliability (Cronbach’s alpha = 0.90) and validity [31]. A Korean community-based study identified an optimal SGDS-K cut-off score of 8 or higher for the screening of major depressive disorders [31].

Other covariates. Since the resources of older adults may influence the types of support and exchange patterns, the analysis was controlled for the variables, reflecting the resources of participants in terms of finance, health and demographic availability, and the possible covariates of depressive symptoms identified by the results of previous population-based studies: age, educational level, place of residence, equivalent household income, employment status, social participation, number of chronic disease, number of close friends or siblings and relatives and living arrangements. Education level was classified as primary school or below, middle school or high school and above. Place of residence was categorized as “urban” or “rural”. Employment status was classified as “yes” or “no”. Equivalent household income was used as a measure of annual income. The total household income was divided by the square root of the number of household members and then categorized by the tertile (lowest, middle or highest 33.3%). Social participation was assessed by asking whether participants engaged in friendships, hobbies, leisure-time activities or political societies. A “yes” response to any social activity was considered indicative of social participation. The number of chronic diseases was noted for all participants. Participants self-reported any physician-diagnosed conditions, including hypertension, stroke, hyperlipidemia, angina pectoris, diabetes, thyroid disease, arthritis, osteoporosis, back pain, sciatica, chronic obstructive pulmonary disease, asthma, tuberculosis, cancer, hepatitis, liver cirrhosis, chronic renal failure, benign prostate hyperplasia, urinary incontinence, sexually transmitted infection, cataracts, glaucoma, chronic otitis media, anemia and chronic dermatologic disease. Living arrangements were classified based on whether participants were living with others (i.e., partner, adult children) or living alone. Living arrangements were categorized as: (1) living with a partner only, (2) living with adult children, (3) living with others and (4) living alone. Social networks were assessed using the question “How many close friends (or siblings and relatives) do you have?” The response options were “none”, “one”, or “two or more”.

### 2.3. Statistical Analyses

The data were expressed as frequencies, weighted proportions or means (± standard deviation (SD)) for the baseline indices of health and socioeconomic status, as well as the types and exchange patterns of intergenerational support (by gender). The distributions of factors were compared using chi-squared tests (Table 1). Logistic regression analyses were used to assess the associations of the types and exchange patterns of each support with depressive symptoms in older adults (Table 2). Models showed the effect of exchange patterns in each type of intergenerational support on depressive symptoms when all covariates were controlled by gender. All results were reported separately for older men and older women. To examine the differences between genders, we performed statistical tests comparing the logit coefficients of gender-specific models [32] with the following steps. We calculated the Wald chi-square statistics to test the differences in the coefficients across gender groups. Then, we adjusted the disturbance variance unconstrained models to assess whether there was significant residual variation between men and women. No significant collinearity was detected between any of the covariates. All statistical analyses were conducted using IBM SPSS software for Windows (ver. 23.0; IBM Corp., Armonk, NY, USA). This study was approved by the Ethics Review Board of Mokpo National University (MNURB-20200120-SB-001-01).

## 3. Results

Demographic characteristics, types and exchange patterns of support and the prevalence of depressive symptoms are presented in Table 1. The mean age of older women (72.24 years) and older men (72.94 years) was similar. A higher proportion of older women (29.6%) than older men (9.4%) lived alone (*p* < 0.001), and the percentage of older men (40.4%) with high school education and above was higher than that of older women (19.1%) (*p* < 0.001). Over half of the older men (68.0%) and women (70.4%) lived in urban areas. The majority of older adults (84.8%) reported social participation, but the proportion was higher in older women (87.7%) than older men (81.4%) (*p* < 0.001). Nearly half of all older men (42.5%) were employed and more than one third (39.8%) had no close friends; the respective rates in older women were 29.4% and 35.5%. The majority of older men and women had two or more chronic diseases (61.1% and 74.7%, respectively).

The exchange patterns of intergenerational support differed by gender, with 57.8% of older men and 68.2% of older women reporting reciprocal emotional support with children and 30.3% of men and 19.1% of women reporting no such reciprocal support. The majority of older men (60.1%) and women (66.3%) received financial support from their children. In nearly 60% of men and 50% of women, reciprocal caring support and instrumental support were lacking. Regarding emotional, instrumental and caring support, the percentage of mutual exchange was significantly higher in older women than older men. The percentages of older men and women who only received support from their children were higher than the percentages of those who only provided support to their children, for all four types of intergenerational support.

Of all participants, 14.6% reported depressive symptoms, with the prevalence being higher among older women (16.5%) than older men (12.5%) (*p* < 0.001). The prevalence rate of depressive symptoms in older adults lacking reciprocal emotional support was higher than that of older adults who did experience mutual emotional support. Older women who provided caring and financial support to their children were more likely to report depressive symptoms than those who only received such support from their children. However, older men who provided instrumental, caring or financial support to their children were less likely to report depressive symptoms than those who only received support. The chi-squared tests revealed that only the emotional and financial support exchange patterns were significantly associated with depressive symptoms in older men and women.

The relationships between each type of intergenerational support and depressive symptoms in older men and women, after controlling for socioeconomic and health characteristics, are shown in Table 2. In both older men (OR = 1.49, 95% CI = 1.17–1.90, *p* < 0.01) and women (OR = 1.87, 95% CI = 1.50–2.34, *p* < 0.001), the lack of a mutual exchange of emotional support with their children was associated with an increased risk of depressive symptoms. Although the gender difference in the association was not statistically significant, both providing emotional support to children (OR = 1.96, 95% CI = 1.14–3.36, *p* < 0.01) and receiving it from them (OR = 1.78, 95% CI = 1.36–2.33, *p* < 0.001) were more strongly associated with depressive symptoms in women than men.

Older men who only received financial support were more likely to experience depressive symptoms (OR = 1.81, 95% CI = 1.36–2.42, *p* < 0.001), whereas older women who provided financial support were more likely to experience depressive symptoms (OR = 2.82, 95% CI = 1.21–6.56, *p* < 0.05). The risk of depressive symptoms in older men lacking the mutual exchange of financial support (OR = 3.83, 95% CI = 2.34–6.24, *p* < 0.001) was higher than that in older women (OR = 1.73, 95% CI = 1.06–2.83, *p* < 0.05). Significant gender differences were observed in the rate of mutual exchange of financial support, based on the Wald chi-square logit coefficients, comparing the logit coefficients across the groups [32].

## 4. Discussion

This study examined the exchange patterns of intergenerational support, and their impact on depressive symptoms, among older men and women in Korea. Gender differences were observed in the exchange patterns for all four types of intergenerational support, and the impact of financial support exchange on depressive symptoms differed by gender.

As expected, older women showed significantly higher rates of providing and receiving individually (and both providing and receiving) all four types of intergenerational support (emotional, instrumental, caring and financial) compared to older men. This finding is supported by previous studies reporting that older women experience more intergenerational contact and support than older men [17,33]. The exchange of financial support with adult children was the most common of the four types of intergenerational support in both older men and older women. The frequency of financial support exchange in this study was higher than that reported by the five-country (Norway, England, Germany, Spain and Israel) study [12]. In addition, the exchange patterns of financial support were markedly different between older adults in Korea and those living in the five western countries in the OASIS study. In Korea, the majority of older men and women receive financial support from their children, whereas older adults in the five western countries were more likely to provide financial support to their children [12]. The country-level differences in financial support exchange, and the higher percentage of older Korean men and women receiving financial support, may be attributed to differences among countries in the welfare systems supporting older adults and their families. According to the Organization for Economic Cooperation and Development’s (OECD) social expenditure database [34], public social welfare spending in Korea in 2013 was 10.4% of the GDP, which was much lower than that of all five countries in the OASIS study (ranging from 16.1% in Israel to 25.3% in Germany). Similar to the finding of a high rate of mutual emotional support among older adults in Korea, the higher percentage of older adults receiving financial support from their children may be explained by the strong filial norms that characterize Korean society. Despite changes to the traditional family structure in East Asia, filial norms remain strong, and intergenerational support typically flows from adult children to their parents [35]. A recent Korean study found that more than 90% of middle-aged children provided emotional and financial support to their parents [36], and other studies found that adult children in Chinese, Korean and Taiwanese families provided more assistance to their parents than they received from them [35]. In this study, the main direction of the exchange of instrumental and caring support was from adult children to their parents. Despite all older adults in Korea having long-term care insurance since 2008 (to reduce the burden of family care), this study found that the percentages of older adults receiving instrumental and caring support from their adult children were 30.5% and 25.7%, respectively. These rates are much lower than those reported in a recent study, which found that about 65% of middle-aged adult children provided instrumental and caring support to their elderly parents [36]. This difference may be attributed to the greater physical independence of the participants of this study; we excluded older men and women with physical limitations and 65.5% of the participants were under the age of 74 (and thus were likely to require less support from their children) (Table 1).

In this study, the lack of a mutual exchange of intergenerational financial support increased the risk of depressive symptoms by 3.58 times for men and 1.66 times for women. Gender differences in the impact of financial support exchange were even greater when comparing the cases of receiving support only and providing support only. Receiving support only was detrimental in older men, whereas providing support only was detrimental in older women. This finding may be explained by the traditional gender roles in Korea, where men are typically the heads of households and assume financial responsibility for the family [37]. When older men receive financial support from their children, it may violate their traditional breadwinner role, leading to feelings of powerlessness and an increased psychological burden [38]. In addition, unlike older women, the older men in our study who only provided financial support had a lower risk of depressive symptoms than those who only received support. When there was no exchange of financial support, older men had an increased risk of depressive symptoms compared to older women. Older Korean men whose entire lives have been lived according to gendered family roles may have less intimate relationships with their children, as their role mainly involves making money and providing for the family. The transfer of emotional and instrumental support typically involves mothers rather than fathers in Korea [39]. These traditional family dynamics may render older Korean men more sensitive to mutual financial support, and they may also be less familiar with other types of mutual support with their adult children. Moreover, the older women in our study who only provided financial support were more likely to have depressive symptoms than those who experienced no exchange at all, which may reflect the financial burden that older women often experience. Traditionally, as homemakers, women provide various types of support associated with rearing and caring for their children throughout their lives. Thus, older Korean women may have limited financial resources, which exacerbates the psychological distress associated with transferring financial resources to their adult children. According to the 2013 report of the Korea Institute for Health and Social Affairs and the Korean Women’s Development Institute, the poverty rate among older men was 40.1%, compared to 45.9% in older women [40]. In addition, 40% of older men had previously earned an income, compared to 15.8% of older women [40]. In 2018, 57.2% of older men benefitted from a national pension, compared to only 29.9% of older women [41].

Although the gender difference was not statistically significant, the impact of unidirectional emotional support on depressive symptoms was greater in women than men. Compared to the mutual exchange of emotional support, the lack of an exchange of emotional support increased the risk of depressive symptoms by 1.42 times for men and 1.74 times for women. In addition, only receiving or only providing emotional support increased the risk of depressive symptoms in women, but not in men. This finding is consistent with previous studies that reported the positive effects of mutual emotional support on the subjective health of older women, but not older men [1], and on the mental health of older adults [42,43]. In addition, other studies have shown that reciprocal support improves the psychological well-being of older adults more so than unidirectional support [8,44]. The negative impact of unidirectional emotional support on depressive symptoms among women may be explained by the strong intergenerational emotional connection among women. Compared to men, women who have stronger ties with their children report more tension, as well as greater emotional closeness, with their children [45]. Therefore, women may experience increased stress when only receiving or only providing emotional support, compared to the case of mutual support.

The effect of instrumental and caring support exchanges on depressive symptoms in older adults was insignificant in this study, unlike previous research which showed that instrumental and caring support is associated with depressive symptoms [46,47]. Our results may partly reflect the competence of the study participants in completing instrumental and caring tasks on their own, because the older adults in this study were free of physical limitations. Instrumental and caring support can be helpful in maintaining mental and physical health under stressful conditions such as declined health and limitation of activities [48]. Meanwhile, older adults who do not have the need for instrumental and caring support can experience a loss of autonomy and a sense of incompetence when they receive instrumental and caring support [49]. The moderation effect of the physical needs of older adults on the association between instrumental and caring support exchange and depressive symptoms should be examined in further studies.

There were several limitations to this study. Firstly, the amount of intergenerational support provided and received was not quantified. Measuring the relative amounts of support provided and received would enable a more detailed analysis of the reciprocal balance of support. Secondly, this study included only the socioeconomic resources of older adults but not those of adult children; resources including the gender, socioeconomic status, marital status and living arrangements of the children were not included as control variables. These reflect adult children’s availability, which may influence the types and exchange patterns of intergenerational support [50]. Lastly, only cross-sectional data were analyzed, which precluded inferences regarding causality. An analysis of the longitudinal data is needed in order to confirm that there are gender differences in the relationships between the types of intergenerational support and depressive symptoms among older adults. Despite these limitations, this study had some notable strengths. Firstly, the data were nationally representative and were weighted based on census estimates, thereby increasing the generalizability of the findings. Secondly, since mobility could impact the need for assistance, and the types and exchange patterns of support, older adults with physical limitations were excluded and it was assumed that participants were sufficiently active to support their children. The results of this study are likely to accurately reflect the exchange patterns of support between older adults and their adult children in Korea. Lastly, this study considered the dual role played by older adults, as both providers and receivers of support, and highlighted the gender differences in the relationships between the different types of support and depressive symptoms among older adults in Korea.

## 5. Conclusions

This study provides evidence of gender differences in intergenerational support exchange and the impact thereof on depressive symptoms, and it suggests that gender differences in intergenerational support exchange and their impact on mental health may differ across diverse cultures. We call for further research evidence from diverse cultural societies, especially from Asian countries, to prove our findings.

## Figures and Tables

**Table 1:** Distribution of intergenerational social support exchange and prevalence of depressive symptoms among older men (*n* = 3592) and older women (*n* = 3939) in the 2017 Korean Living Profile Survey of Older People.

	Older Men		Older Women		*P*	All		Prevalence of Depressive Symptoms (% or Mean ± SD)			
								Older Men	Older Women	*P*	All
	*N*	Weighted %	*N*	Weighted %		*N*	Weighted %				
*N=*	3592		3939			7531		12.5	16.5	*p* < 0.001	14.6
								2.96 ± 3.45	3.54 ± 3.73		3.27 ± 3.62
Emotional support exchange								**	**		**
No exchange	1082	30.3	755	19.1	*p* < 0.01	1837	24.3	18.4	25.4	*p* < 0.001	21.3
Receiving support only	316	8.8	389	10.3		705	9.6	14.0	25.1		20.3
Providing support only	112	3.1	98	2.4		210	2.7	10.1	21.9		15.5
Mutual support	2082	57.8	2697	68.2		4779	63.4	9.2	12.5		11.1
Instrumental support exchange											
No exchange	1838	51.4	1584	40.7	*p* < 0.01	3422	45.7	12.5	17.7	*p* < 0.001	14.8
Receiving support only	1142	31.7	1121	29.3		2263	30.5	12.8	15.7		14.3
Providing support only	171	4.7	306	7.3		477	6.1	8.1	14.0		11.9
Mutual support	441	12.1	928	22.6		1369	17.7	13.2	16.7		15.5
Caring support exchange											
No exchange	2596	72.5	2406	61.1	*p* < 0.01	5002	66.4	12.0	16.5	0.031	14.2
Receiving support only	767	21.2	1157	29.7		1924	25.7	14.3	16.6		15.7
Providing support only	39	1.1	64	1.6		103	1.3	9.8	22.7		17.9
Mutual support	190	5.2	312	7.7		502	6.5	12.7	14.7		13.9
Financial support exchange								**	**		**
No exchange	272	4.5	205	3.0	*p* < 0.01	477	3.7	33.7	32.6	0.011	33.2
Receiving support only	2199	60.1	2663	66.3		4862	63.4	14.4	17.9		16.3
Providing support only	41	1.4	32	0.9		73	1.2	8.7	24.2		15.4
Mutual support	1080	34.0	1039	29.7		2119	31.7	6.5	11.4		8.9
Age (Mean ± SD)	72.94 ± 5.87		72.24 ± 5.63			72.57 ± 5.76		**	**		**
65–74	2271	63.4	2714	67.2	*p* < 0.01	4985	65.5	11.1	14.8	0.176	13.1
74–85	1165	32.7	1100	29.5		2265	31	15.4	20.8		18.1
85 and over	156	3.9	125	3.3		281	3.5	11.6	16.0		13.6
Place of residence								**	**		
Urban	2367	68.0	2663	70.4	0.127	5031	69.2	13.1	16.7	0.784	15.1
Rural	1225	32.0	1276	29.6		2500	30.8	10.9	15.6		13.4
Education								**	**		**
High school and over	1451	40.1	753	18.5	*p* < 0.01	2204	28.6	9.0	10.0	*p* < 0.001	9.3
Middle school	778	21.7	662	16.5		1440	18.9	12.0	11.5		11.7
Primary school and less	1363	38.2	2524	64.9		3887	52.4	16.7	19.8		18.7
Equivalent household income								**	**		**
1st 33.3% (highest)	1451	40.1	1346	32.6	*p* <0.001	2797	36.1	7.1	10.2	0.753	8.6
2nd 33.3%	1192	33.2	1364	34.6		2556	34.0	12.5	16.0		14.4
3rd 33.3%	949	26.7	1229	32.7		2178	29.9	20.8	24.0		22.6
Living arrangements								**	**		**
Living alone	335	10.5	1167	32.6	*p* < 0.001	1502	22.3	16.1	13.2	*p* < 0.001	13.8
Living with partner only	2345	64.7	1687	41.4		4032	52.3	5.0	7.0		5.9
Living with adult-children	782	21.3	955	22.9		1737	22.1	7.0	10.6		9.0
Living with others	130	3.5	129	3.1		259	3.3	7.7	11.6		9.7
Number of chronic diseases								**	**		**
None	590	16.5	374	9.4	*p* < 0.001	964	12.7	7.3	3.5	*p* < 0.001	5.8
One	808	22.4	621	15.7		1429	18.8	7.3	7.2		7.2
Two and over	2194	61.1	2944	74.9		5138	68.5	15.9	20.1		18.3
Number of friends in close contact								**	**		**
None	1430	39.7	1413	35.9	*p* < 0.001	2843	37.6	18.2	25.3	0.066	21.7
One	622	17.4	847	21.6		1468	19.7	11.3	16.2		14.1
Two and over	1540	42.9	1679	42.5		3219	42.7	7.8	9.3		8.6
Number of siblings and relatives in close contact								**	**		**
None	1962	54.6	1811	46.2	*p* < 0.001	3773	50.1	16.0	22.8	0.105	19.2
One	845	23.6	1174	29.9		2019	26.9	10.3	12.8		11.7
Two and over	785	21.9	954	23.9		1739	23.0	6.4	9.2		7.9
Social participation								**	**		**
Yes	2950	81.4	3467	87.7	*p* < 0.001	6417	84.8	10.4	14.6	*p* < 0.001	12.7
No	642	18.6	472	12.3		1114	15.2	21.8	30.1		25.3
Working status								**	**		**
Yes	1526	42.6	1160	29.9	*p* < 0.001	2686	35.8	7.1	11.7	0.257	9.1
No	2066	57.4	2779	70.1		4845	64.2	16.6	18.5		17.7

* *p* < 0.05; ** *p* < 0.01 for differences among the levels of each variable. SD: standard deviation.

**Table 2:** Adjusted odds ratios (ORs) and 95% confidence intervals (CIs) for depressive symptoms among older men (*n* = 3592) and older women (*n* = 3939) in the 2017 Korean Living Profile Survey of Older People.

	Older Men				Older Women			
	Model 1	Model 2	Model 3	Model 4	Model 1	Model 2	Model 3	Model 4
Emotional support exchange								
No exchange	1.49 (1.17–1.90) **				1.87 (1.50–2.34) **			
Receiving support only	1.17 (0.81–1.69)				1.78 (1.36–2.33) **			
Providing support only	1.34 (0.68–2.65)				1.96 (1.14–3.36) **			
Mutual support (reference)	1				1			
Instrumental support exchange								
No exchange		0.76 (0.49–1.16)				0.95 (0.67–1.34)		
Receiving support only		0.84 (0.55–1.29)				0.75 (0.52–1.08)		
Providing support only		0.58 (0.31–1.10)				1.14 (0.80–1.66)		
Mutual support		1				1		
Caring support exchange								
No exchange			0.71 (0.44–1.16)				1.25 (0.86–1.82)	
Receiving support only			0.84 (0.50–1.40)				1.07 (0.73–1.57)	
Providing support only			0.65 (0.20–2.14)				1.61 (0.79–3.28)	
Mutual support			1				1	
Financial support exchange †								
No exchange				3.83 (2.34–6.24) **				1.73 (1.06–2.83) *
Receiving support only				1.81 (1.36–2.42) **				1.19 (0.95–1.48)
Providing support only				1.10 (0.32–3.79)				2.82 (1.21–6.56) *
Mutual support				1				1
Adjusted *R^2^*	0.155	0.152	0.151	0.166	0.185	0.171	0.171	0.173
Hosmer & Lemeshow *(p-value)*	0.992	0.977	0.988	0.805	0.964	0.329	0.482	0.873

Adjusted by age, educational level, place of residence, equivalent household income, living arrangements, number of chronic diseases, number of friends/siblings and relatives in close contact, social participation and working status. † *p* < 0.05 by Wald chi-square statistics for testing the differences between coefficients for men and women; * *p* < 0.05, ** *p* < 0.01.
